# Renal replacement therapy for children throughout the world: the need for a global registry

**DOI:** 10.1007/s00467-017-3863-5

**Published:** 2017-12-22

**Authors:** Sophie Ploos van Amstel, Marlies Noordzij, Bradley A. Warady, Francisco Cano, Jonathan C. Craig, Jaap W. Groothoff, Kenji Ishikura, Alicia Neu, Hesham Safouh, Hong Xu, Kitty J. Jager, Franz Schaefer

**Affiliations:** 1 IPNA Global RRT Registry, Department of Medical Informatics, Academic Medical Center, Amsterdam Public Health research institute, University of Amsterdam, PO Box 22700, 1100 DE Amsterdam, The Netherlands; 2 Division of Nephrology, Children’s Mercy Kansas City, University of Missouri-Kansas City School of Medicine, Kansas City, MO USA; 3 Division of Pediatrics, Luis Calvo Mackenna Children’s Hospital, Faculty of Medicine, University of Chile, Santiago, Chile; 4 Department of Nephrology, Children’s Hospital at Westmead, School of Public Health, University of Sydney, Sydney, Australia; 5 Department of Pediatric Nephrology, Emma Children’s Hospital AMC, University of Amsterdam, Amsterdam, The Netherlands; 6 Division of Nephrology and Rheumatology, National Center for Child Health and Development, Tokyo, Japan; 7 Division of Pediatric Nephrology, The Johns Hopkins University School of Medicine, Baltimore, MD USA; 8 Pediatric Nephrology Unit, Faculty of Medicine, Cairo University, Cairo, Egypt; 9 Kidney Development & Pediatric Kidney Disease Research Center, Children’s Hospital of Fudan University, Shanghai, China; 10 Division of Pediatric Nephrology, Heidelberg University Center for Pediatrics and Adolescent Medicine, Heidelberg, Germany

**Keywords:** Registry, Epidemiology, Renal replacement therapy, Dialysis, Kidney transplantation

## Abstract

**Background:**

To describe the factors affecting the incidence of renal replacement therapy (RRT) among children, information from RRT registries is required. We aimed to give an overview of existing pediatric RRT registries worldwide, identify regions with a need to commence or increase data collection on pediatric RRT, and provide a rationale for developing a global RRT registry.

**Methods:**

A survey assessing pediatric RRT registry status was sent to International Pediatric Nephrology Associateion (IPNA) members in 127 countries in January 2016. The survey was complemented by a systematic literature search for active pediatric RRT registries.

**Results:**

Complete survey responses were retrieved from 94 countries (representing 86.2% of the world childhood population), with 84 (81.2%) having the means to provide RRT to children, given that there are no other limitations such as financial, social, or religious restraints. Fifty-one (35.3%) countries had national registries for both dialysis and transplantation, nine (30.0%) either had a dialysis or a transplant registry, six participated in international registries only (2.7%), and in 18 (13.2%), children on RRT were not followed in any registry. The search identified 92 pediatric RRT registries, primarily national registries located in Europe, North America, and Asia.

**Conclusions:**

Although pediatric RRT can be provided in 84 countries representing 81.2% of the world’s childhood population, national pediatric RRT registries are unavailable in many countries. To improve knowledge about the incidence and outcomes of pediatric RRT around the globe, an international population-based pediatric RRT registry has recently been initiated.

**Electronic supplementary material:**

The online version of this article (10.1007/s00467-017-3863-5) contains supplementary material, which is available to authorized users.

## Introduction

Renal replacement therapy (RRT) is a life-saving, high-cost treatment that requires expertise and sustained funding. Whereas RRT has been available for children in developed countries for almost 50 years, it has only recently been introduced or is still largely unavailable in many developing countries [1]. The incidence rates of RRT in children vary considerably on the global scale, from four per million age-related population (pmarp) in developing nations to 14 pmarp in developed countries [2]. The variation in incidence rates is believed to be largely explained by differences in national wealth and health expenditure. However, even within countries with comparable wealth and health expenditure, variation in RRT incidence is present [2, 3].

In addition, differences in healthcare organization and delivery at a national level, ethnic differences in disease prevalence and sociocultural variation in the approach towards congenital and chronic disease in children may add to the observed variation in incidence rate and access to RRT. Information from RRT registries offers the potential of a better understanding of the factors affecting pediatric RRT incidence rates at an ecological level [4, 5]. Moreover, clinical quality RRT registries may improve the quality of care, since in addition to incidence and prevalence rates, they reveal data on practice patterns and outcomes [5, 6].

Consequently, most developed countries have established national pediatric RRT registries, either as stand-alone databases or integrated into adult renal registries. However, it is well recognized that population-based national renal registries of adult patients are still unavailable for countries that altogether comprise at least half of the world’s population [7]. This leaves a large part of the global end-stage renal disease (ESRD) population unidentified and their caregivers and health care providers uninformed about the size of the treatment challenges ahead. Similarly, current data regarding the global availability of pediatric RRT registries are lacking.

To initiate global collaboration and to help create pediatric RRT registries in countries without such a registry, the International Pediatric Nephrology Association (IPNA) has recently started the IPNA Global RRT Registry. This paper aims to: (1) give an overview of existing renal registries reporting on pediatric RRT, (2) identify regions with a need to commence or expand data collection on pediatric RRT, and (3) provide the rationale for the initiation of this global RRT registry.

## Methods

For this article, we combined an Internet-based survey among IPNA members with a systematic literature search for pediatric renal registries to identify existing pediatric RRT registries. We defined a RRT registry as a systematic collection of a clearly defined set of health and demographic data for patients on RRT, held in a central database [5]. All data generated or analyzed during this study are included in this published article and its supplementary information files.

### Survey

**Fig. 1:**
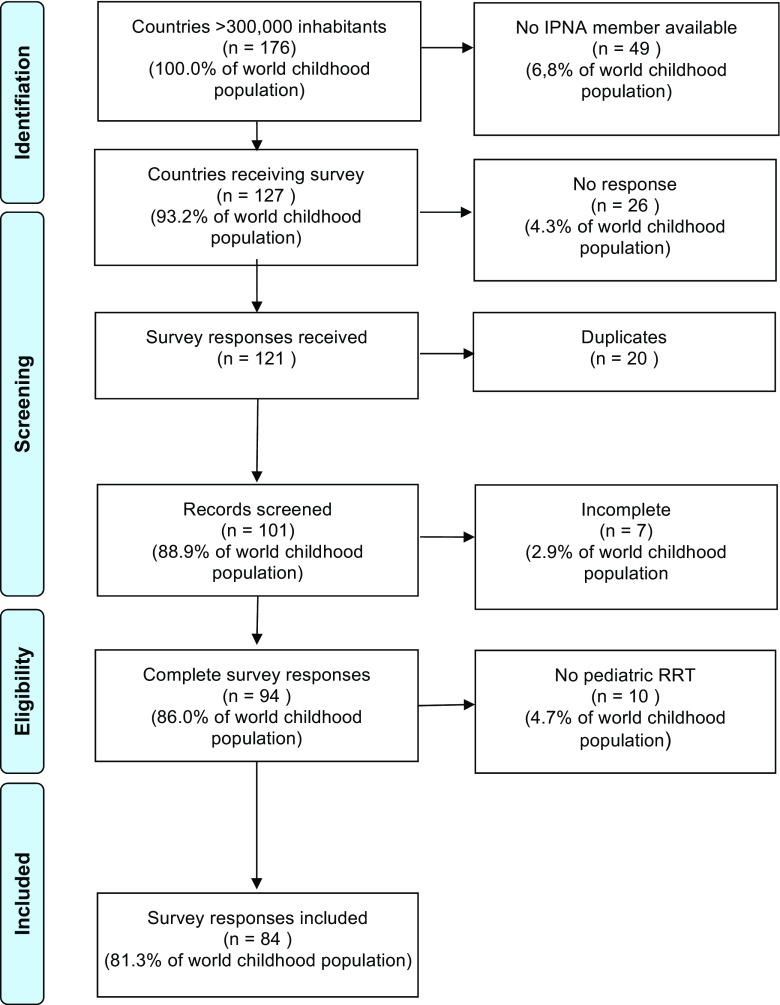
Flow chart of the survey

We identified a total of 176 countries with more than 300,000 inhabitants (representing 99.9% of the world’s population). In 127 of these 176 countries, representing 93.2% of the global childhood population under 15 years of age, an IPNA member was identified and contacted (see Fig. 1). In the period from January to March 2016, the survey was electronically distributed. No paper surveys were sent. If a country had more than one IPNA contact, the individual believed to have the greatest expertise in RRT registries was selected. If a contact believed that a colleague was more suitable to complete the survey, the survey could be forwarded. If multiple responses from one country were received and responses were discrepant, both respondents were contacted and consensus was reached. Surveys were not sent to the 49 countries (representing 6.8% of the global childhood population) without an IPNA member. These gaps were located in Africa (*n* = 24), Latin America (*n* = 9), Asia (*n* = 15), and Europe (n = 1).

The survey comprised four closed and five open-ended questions assessing the estimated numbers of prevalent chronic pediatric RRT patients, treatment modalities, and the registry status of each individual country (see Online Resource 1). Question response types included multiple-choice and free text responses. Reminders were sent three and six weeks after the initial survey was distributed. The survey closed in November 2016. Information on population size was collected from the World Bank [8]. As we did not use or collect any patient identifiable data, ethical approval was not required.

### Literature search

An electronic search was performed to identify registries of children with ESRD receiving RRT. The search strategy was based on the strategy used in a recent systematic review describing adult renal registries around the world [7]. The search terms used were combined with fields referring to pediatric data (see Online Resource 2). All human studies identified were included without date or language restrictions. Titles and abstracts were screened to identify articles that could potentially meet the inclusion criteria, which was confirmed by full-text review. Electronic searches for pediatric renal registry websites were also performed. If the website or obtained article was in a language other than English or Dutch, Google Translate was used for translation. Finally, the reference lists of all eligible articles, including review articles, were manually reviewed for potential additional information regarding pediatric renal registries.

Registries meeting the following inclusion criteria were selected: (1) inclusion of pediatric data (less than 21 years of age), (2) data on chronic RRT (dialysis and/or kidney transplantation), and (3) active in the past 10 years (i.e., 2006–2016). Cohort studies, birth registries, and registries on renal biopsies, rare diseases, or acute kidney injury only were excluded. Registries were also excluded if there was no publically available information on the registry website or in published articles to determine the scope of the registry. No attempts were made to establish contact with the registry personnel. Survey respondent data were reported as counts and proportions for all categorical variables, and as absolute values for continuous variables.

## Results

### Survey

#### Response

Surveys were returned by respondents from 101 countries (response rate 80%), comprising 88.9% of the world’s childhood population (see Fig. 1). Respondents identified themselves as pediatric nephrologists (60%), nephrologists (14%), researchers (12%), pediatricians (3%), registry staff (2%), or not specified (9%). The 26 countries from which no response was received (representing 4.3% of the global childhood population) were located in Africa (*n* = 7), Latin America (*n* = 4), Asia (*n* = 6), and Europe (*n* = 9). Childhood population coverage by survey respondents varied by continent, from 73.4% in Africa to 99.7% in Northern America and Oceania. Of the 101 country responses, seven were excluded from further analysis because of incompleteness, leaving 94 for analysis, which represented 86.0% of the world’s childhood population. The list of countries and their response status is given in the supplementary material (See Online Resource 3.1).

#### Provision of RRT care

**Fig. 2:**
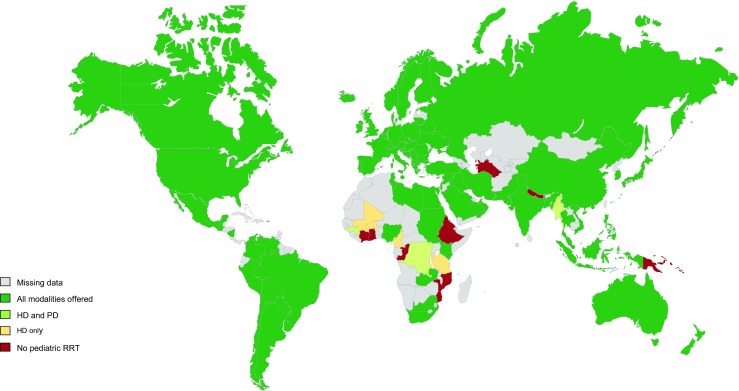
Countries were pediatric renal replacement therapy is offered according to the survey. *RRT* renal replacement therapy, *Tx* transplantation, *HD* hemodialysis, *PD* peritoneal dialysis

**Fig. 3:**
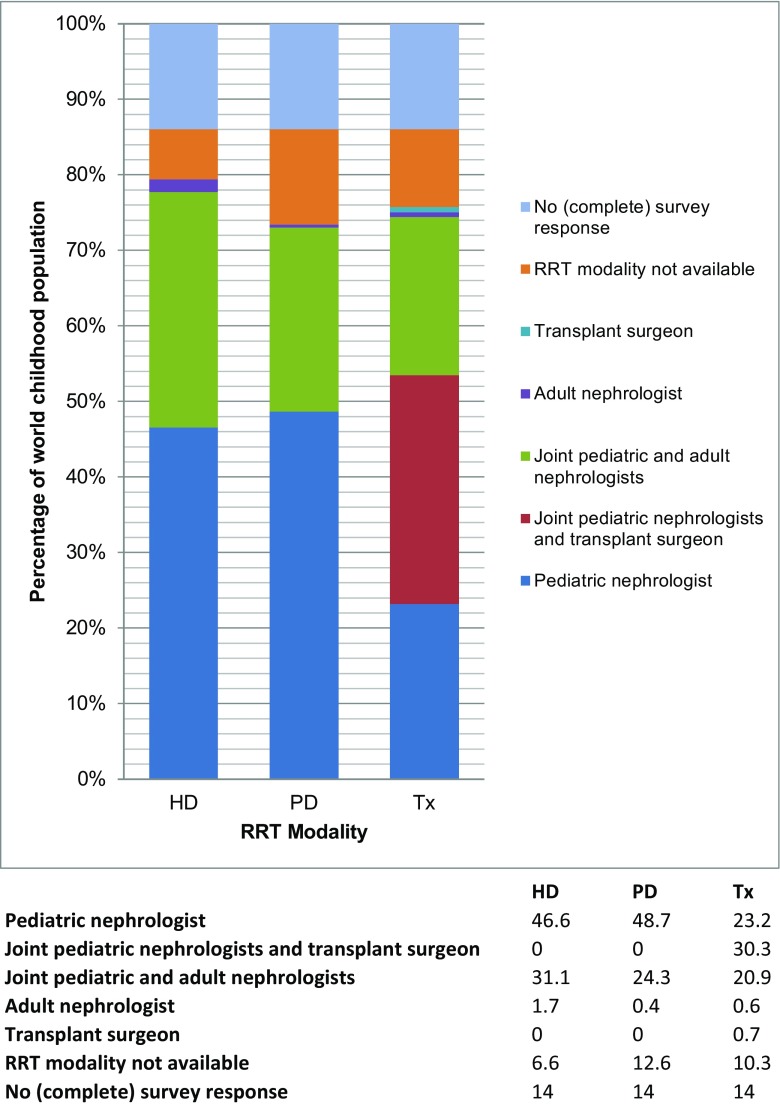
Specialist in charge of treatment by RRT modality, given as the percentage of the world's childhood population, based on 94 complete survey responses

Of the 94 complete responses, ten contacts indicated that there was no chronic pediatric RRT in their country (Ethiopia, Ghana, Nepal, Mozambique, Côte d’Ivoire, Papua New Guinea, Turkmenistan, Republic of Congo, Fiji, and the Solomon Islands—together representing 4.7% of the world’s childhood population) (see Fig. 2). In 35 countries with pediatric RRT (representing 59.7% of the world’s childhood population), children are cared for by pediatric nephrologists in combination with adult nephrologists or transplant surgeons. In 43 countries (20.0%), children are primarily taken care of by pediatric nephrologists alone. In four countries (1.6%; Burkina Faso, Zambia, Guinea, and Burundi), pediatric nephrologists are lacking and pediatric patients are cared for by adult nephrologists (see Fig. 3). In two countries (0.7%) (Ukraine and Colombia), pediatric transplant recipients are referred to the transplant surgeon for long-term care.

#### Existing registries as reported by IPNA members

**Fig. 4:**
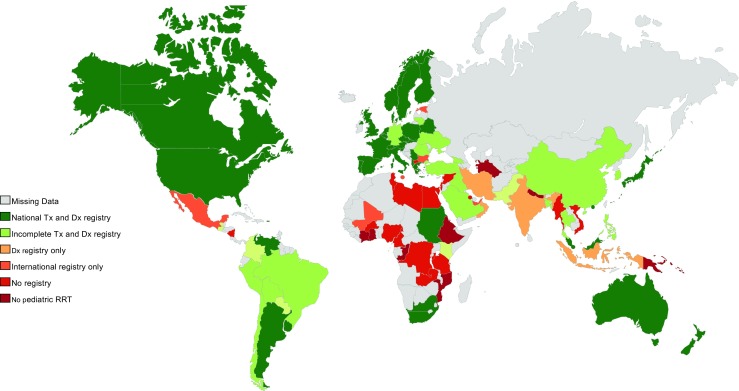
Countries with national registries in place according to survey. *Tx* transplantation, *Dx* Dialysis, *RRT* renal replacement therapy

Of the 84 countries where chronic pediatric RRT is available, 49 (58.3%) representatives indicated that national registries for both dialysis and transplantation are in place. One-third of the registries (*n* = 17) may be incomplete, as some but not all patients are followed. Five country representatives (6.0%) indicated that they only have a national dialysis registry and five (6.0%) reported that they have a national transplant registry only. A total of 18 responses (21.4%) indicated there were no national registries for pediatric RRT in their country. In six (7.1%) countries, data were contributed to international registries only, and one (1.2%) had a national peritoneal dialysis (PD) registry and contributed to an international transplant registry. As shown in Fig. 4, in Europe and North America, almost all countries have national registries. In contrast, population-based pediatric RRT registries are lacking in large parts of Africa, Asia, and Latin America.

### Systematic literature search: Characteristics of registries

The literature search yielded 380 references that included at least one data element suggesting pediatric renal registry activity. After screening titles and abstracts, 85 remained. Manual Internet searches resulted in information relevant to seven additional registries. In total, 92 pediatric RRT registries were identified.

**Fig. 5:**
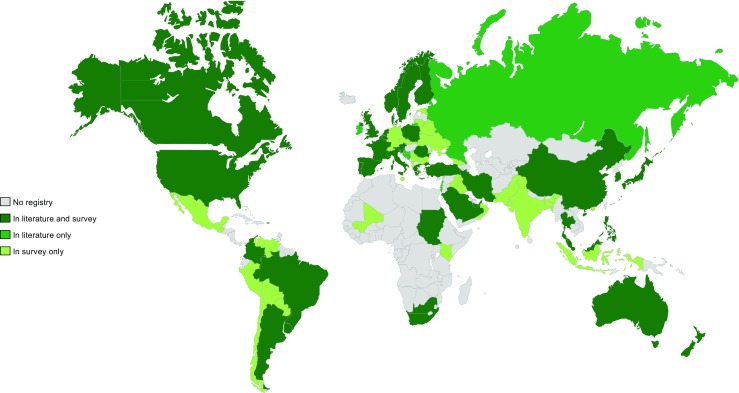
Countries where a national pediatric renal replacement therapy (RRT) registry is present according to literature and/or survey

Most registries (*n* = 67, 72.8%), were organized on a national level. Twelve smaller registries (13.0%) were organized on a regional level (i.e., a province or a region of a country) and 13 (14.1%) larger registries were international. Of the national pediatric RRT registries, 11 (16.4%) reported on dialysis only, 13 (19.4%) reported on kidney transplantation only, and 43 (64.2%) reported on both dialysis and transplantation. Those registries were primarily located in Europe, the Asia/Pacific region, and North America (Fig. 5).

In the supplementary material (see Online Resource 3.2 and 3.3), an overview is given of all national pediatric RRT registries that were identified either via our survey or via the literature search. Seven RRT registries (in Israel, Lebanon, Brunei Darussalam, Russia, Ireland, Croatia, and Bosnia and Herzegovina) were identified through literature search only because of the absence of an IPNA member contact or the lack of response to the online survey (see Online Resource 3.4). For 19 countries, located primarily in Eastern Europe and Asia, the existence of a pediatric dialysis registry was mentioned in the survey response, but could not be tracked by literature/Google search. Survey respondents from 21 (mostly Eastern European and Latin American) countries indicated the existence of national pediatric transplant registries which were not identified in the literature search.

### International registries

Ten international registries and four inter-continental registries were identified (see Online Resource 3.5). Two international registries reported data on dialysis only, four on transplantation only, and four registries included both transplantation- and dialysis-related data. The four intercontinental registries were the International Pediatric Dialysis Network (IPDN), the International Quotidian Dialysis Registry (IQDR), the Collaborative Transplant Study (CTS), and the Cooperative European Pediatric Renal Transplant Initiative Registry (CERTAIN). IPDN was the largest dialysis registry and the CTS was the largest transplantation registry. The largest population-based continental RRT registries were the ones organized by the Asociación Latinoamericana Nefrología Pediátrica (ALANEPE) and the European Society Pediatric Nephrology/European Renal Association – European Dialysis Transplantation Association (ESPN/ERA-EDTA).

Seven country representatives (8.3%) indicated that all their hemodialysis (HD) patients are followed in the IPDN, and only one (Mali) indicated that there was no national registry. In ten countries (11.9%), all the pediatric peritoneal dialysis (PD) patients are followed in the IPDN, and only one of them (Macedonia) indicated that there was no national registry. For transplantation, all patients from 24 countries (28.6%) are followed in an international registry, whereas in ten countries (11.9%), some, but not all, patients are followed in international registries.

### Discussion

According to the survey respondents from 94 countries, chronic pediatric RRT exists in 84 countries, but is absent in ten. However, it should be emphasized that the existence of pediatric RRT in a country does not guarantee access of all children to RRT. For instance, in India and China, the world’s largest countries encompassing 32% of the global childhood population, the estimated prevalence of pediatric RRT is less than 10% of that observed in Western countries (pers. comm.). Reported pediatric RRT availability merely means that basic facilities for performing chronic pediatric RRT do exist in such country. In less-developed countries, the actual accessibility to chronic RRT for children is often importantly hampered by the paucity of RRT sites and financial hurdles [9]. As chronic RRT requires sustained, high-level funding and many patients in developing countries do not have insurance or sufficient income to pay for RRT directly, chronic RRT is often not feasible. Furthermore, in addition to the financial constraints, some patients may also struggle with various legal, social, and religious obstacles to RRT [9, 10].

Ten countries, representing 4.3% of the global childhood population, apparently do not offer chronic pediatric RRT at all. In five of these countries, all located in Africa, respondents indicated that there are no pediatric nephrologists and patients with acute kidney injury and in need of acute dialysis are taken care of by adult nephrologists. In many developing countries, adult nephrologists appear to play an important role in pediatric RRT. This is likely related to the fact that pediatric RRT centers are commonly located in urban areas, often far from where children with ESRD are physically located, but in proximity to rural adult dialysis centers [9]. We can, in turn, speculate that the absence of a local pediatric nephrologist results in the limited provision of pediatric RRT, in particular to younger children, as adolescents are more frequently treated by adult nephrologists.

Given that the mean GDP in most of the 49 countries without an IPNA member (representing 6.8% of the world’s children) was similar or lower than that of the countries with a documented complete lack of chronic RRT, these countries should probably be added to the number of countries without chronic pediatric RRT. The impact of national GDP differences on the variation of RRT incidence across countries has recently been demonstrated for the European continent [3]. Macroeconomic differences limit the provision of RRT to the youngest children in particular, with only the wealthier countries having the capacity to treat pediatric patients of all age groups [3]. Globally, these differences in macroeconomics may be the leading cause of inequalities in access to care, with social and logistic issues playing important additional roles [9, 10].

### Pediatric RRT registries

Population-based national registries of pediatric RRT patients are required to describe the global incidence of pediatric RRT and to capture variations in treatment and outcomes. It appears that most developed countries have national registries for both dialysis and transplantation in place. In contrast, large parts of Africa and the Middle East either do not have a registry at all, or appear to have a registry that is limited to certain regions.

The lack of comprehensive registries in developing countries is plausible because a renal registry requires substantial, long-term investment by payers and health care providers, and also requires a critical mass of administrative/technical staff [4, 5, 7]. In addition to funding, good infrastructure and communication between RRT centers and registry staff (facilitated by reliable internet access) is required. Whereas the concentration of care in large hospitals should facilitate patient monitoring, it may be challenging, especially in very large countries. In such countries, geographic distances and poor infrastructures often result in decentralized care in adult nephrology or non-specialized pediatric units, which could result in incomplete registration of children in national RRT databases and underestimation of the pediatric RRT or ESRD incidence.

Finally, most international registries that do exist are not population-based, meaning that they do not receive data from all pediatric RRT centers. The problem with inclusion of data from registries that are not population-based is that demographic information derived from those sources may be incomplete and potentially biased due to overrepresentation of well-organized centers with a particular interest in RRT [10]. Until now, a global population-based pediatric RRT registry created with patient-level data has been lacking.

### Strengths and limitations

We used a combination of a survey and a systematic literature search to identify pediatric RRT registries. The response rate to the survey was high, with IPNA representatives from 101 out of 127 countries providing information from regions representing 86.2% of the world’s childhood population. This could be achieved because of IPNA’s broad network, comprising over 1500 pediatric nephrologists and allied professionals in more than 100 countries around the world [7]. However, some potential limitations of this analysis should be acknowledged. The combination of the survey and the literature search revealed some minor discrepancies. Some registries were exclusively found in the literature search because we did not receive a response or did not have a contact person in the respective country. Other registries were mentioned in the survey, but could not be found in our electronic searches. The absence of results in our literature search may not necessarily mean that there is no registry activity in those countries as they sometimes do contribute data to international registries. At the same time, some of the national registries may be very difficult to detect when they are operating without publicly available information like a website, periodic reports or journal publications. An additional reason for these discrepancies could be that only one contact person per country completed our survey and answers may therefore not be representative of the entire country. Another possible reason is that respondents were not fully aware of any registry activity in their country. This could have caused misrepresentation of registry status for the entire country, ultimately leading to an underestimation of the number of pediatric RRT registries worldwide.

### The IPNA Global RRT Registry

Our findings are in line with the recent findings of Liu et al. regarding adult RRT registries and support the recommendations that emerged from that study: “*Gaps in registry coverage, data collection, and completeness present an opportunity for more productive collaborations to collect relevant data, implement quality and standardization procedures, and provide broad access to comprehensive information to facilitate the advancement in patient care and research.*” [7] To this end, the IPNA Council recently initiated a global collaborative registry effort in the field of pediatric RRT. The IPNA Global RRT Registry aims to: (1) empower clinical and translational research through information on disease demographics, management, and outcomes; (2) benchmark performance on a country level according to key performance indicators; and (3) facilitate clinical trial planning through the collection of information on available population sizes. Additionally, it is hoped that this global registry will increase recognition of the worldwide problem of pediatric ESRD and RRT, and highlight discrepancies in the access to pediatric RRT services between countries and continents [11–13]. This information could then be leveraged to encourage governments and other funders to make an even greater effort to improve pediatric ESRD care.

Each national or international registry around the globe has been invited to submit data on an annual basis to a core dataset containing patient level data regarding age, sex, primary renal disease, date and modality of RRT, as well as date and cause of death. In addition, direct data submission will be made possible for countries without existing registries provided that complete population coverage is attained. In this way, national pediatric RRT registries will emerge by participation in the international registry. The IPNA Global RRT Registry will use the information obtained to produce reports describing country-specific RRT incidence and prevalence, modality choices, and patient survival rates. Detailed demographic and benchmarking figures will be generated to compare country-specific pediatric RRT characteristics on a regional and global level. Ultimately, collaboration between the IPNA registry and existing adult RRT registries will provide the opportunity to better characterize the long-term outcome of pediatric ESRD patients on a global basis.

### Conclusions

To describe the global incidence of pediatric RRT and to capture variations in treatment and outcomes, registration of pediatric RRT patients is warranted. Comprehensive national pediatric RRT registry databases are still largely unavailable for many developing countries, resulting in fragmented and incomplete knowledge regarding pediatric RRT management around the globe. To overcome these gaps in global registry coverage and strengthen international collaboration, IPNA is launching a global pediatric RRT Registry. Through this large-scale collaborative effort, the collection of information will ideally bring attention to the availability and complexities related to RRT care for children around the world.

## Electronic supplementary material

ESM 1 (PDF 306 kb)

ESM 2 (PDF 304 kb)

ESM 3 (XLS 227 kb)
