# Supplementary material for: Renal replacement therapy for children throughout the world: the need for a global registry
Source: Pediatr Nephrol. 2017 Dec 22;33(5):863–71. doi: 10.1007/s00467-017-3863-5 (PMC5861175; doi:10.1007/s00467-017-3863-5)
Supplement: Supplementary file 1 — (PDF 306 kb) [file 467_2017_3863_MOESM1_ESM.pdf]

Renal replacement therapy for children throughout the world: the need for a global registry

Authors: S. Ploos van Amstel [1], M. Noordzij [1], B. Warady [2], F. Cano [3], J.C. Craig [4], J.W. Groothoff [5], K. Ishikura [6], A. Neu [7], H. Safouh [8], H. Xu [9], K.J. Jager [1], F. Schaefer [10]

[1] IPNA Registry, Department of Medical Informatics, Academic Medical Center, Amsterdam Public Health research institute, University of Amsterdam, Amsterdam, The Netherlands

[2] Division of Nephrology, Dialysis and Transplantation Children's Mercy, University of Missouri-Kansas City School of Medicine, Kansas City, United States of America.

[3] Division of Pediatrics, Luis Calvo Mackenna Children's Hospital, Faculty of Medicine, University of Chile, Santiago, Chile

[4] Department of Nephrology, Children's Hospital at Westmead, School of Public Health, University of Sydney, Sydney, Australia

[5] Department of Pediatric Nephrology, Emma Children's Hospital AMC, University of Amsterdam, Amsterdam, The Netherlands

[6] Division of Nephrology and Rheumatology, National Center for Child Health and Development, 2-10-1, Okura, Setagaya-ku, Tokyo 157-8535, Japan

[7] Division of Pediatric Nephrology, The Johns Hopkins University School of Medicine, Baltimore, The United States of America.

[8] Pediatric Nephrology Unit, Faculty of Medicine, Cairo University, Cairo, Egypt

[9] Kidney Development & Pediatric Kidney Disease Research Center, Children's Hospital of Fudan University, Shanghai, China

[10] Division of Pediatric Nephrology, Heidelberg University Center for Pediatrics and Adolescent Medicine, Heidelberg, Germany

Keywords: *Registry; Epidemiology; Renal replacement therapy; Dialysis; Kidney transplantation.*

Funded by International Pediatric Nephrology Association

Word Count: 3142 (excluding abstract, references, tables and figures)

Word count abstract: 220

Correspondence to:

Sophie Ploos van Amstel, MD

IPNA Registry, Department of Medical Informatics,

Academic Medical Center, University of Amsterdam

P.O. Box 22700, 1100 DE Amsterdam, The Netherlands

Telephone +31 20 566 5738; E-mail: s.ploosvanamstel@amc.uva.nl

SUPPLEMENTARY MATERIAL

SURVEY

1) General information
   Name:
   Position:
   Hospital:
   City/Town:
   State/Province:
   Country:
   E-mailadress:

2) Please provide us with a rough estimate of the total number of pediatric patients currently undergoing renal replacement therapy in your country.
   Number of patients on chronic HD
   Number of patients on chronic PD
   Number of transplant recipients

3) If chronic HD, PD or Tx is offered to children in your country, who is in charge of their treatment? Please choose from the list:
   Mostly or exclusively pediatric nephrologists
   Joint management by pediatric and adult nephrologists
   Mostly or exclusively adult nephrologists
   This treatment is currently not offered to children in my country

   If none of the possible answers to the preceding questions correctly describe the situation in your country, please feel free to add additional comments here.

4) Are pediatric patients on renal replacement therapy currently followed in one or several registries in your country? Please provide answers for the children on HD, PD and Tx respectively. Answers are not exclusive, so please tick all correct answers.

   HD
   No HD patients from my country are currently followed in a registry.
   All HD patients from my country are followed in a national registry.
   Some but not all HD patients from my country are followed in a national or regional registry.
   All HD patients from my country are followed in IPDN.

   PD
   No PD patients from my country are currently followed in a registry.
   All PD patients from my country are followed in a national registry.
   Some but not all PD patients from my country are followed in a national or regional registry.
   All PD patients from my country are followed in IPDN.
   Some but not all PD patients from my country are followed in IPDN.

   TX
   No Tx patients from my country are currently followed in a registry.
   All Tx patients from my country are followed in a national registry.
   Some but not all Tx patients from my country are followed in a national or regional registry.
   All Tx patients from my country are followed in an international registry.
   Some but not all Tx patients from my country are followed in an international registry.

Please feel free to add additional comments here.
