# Supplementary material for: Renal replacement therapy for children throughout the world: the need for a global registry
Source: Pediatr Nephrol. 2017 Dec 22;33(5):863–71. doi: 10.1007/s00467-017-3863-5 (PMC5861175; doi:10.1007/s00467-017-3863-5)
Supplement: Supplementary file 2 — (PDF 304 kb) [file 467_2017_3863_MOESM2_ESM.pdf]

Renal replacement therapy for children throughout the world: the need for a global registry

Authors: S. Ploos van Amstel [1], M. Noordzij [1], B. Warady [2], F. Cano [3], J.C. Craig [4], J.W. Groothoff [5], K. Ishikura [6], A. Neu [7], H. Safouh [8], H. Xu [9], K.J. Jager [1], F. Schaefer [10]

[1] IPNA Registry, Department of Medical Informatics, Academic Medical Center, Amsterdam Public Health research institute, University of Amsterdam, Amsterdam, The Netherlands

[2] Division of Nephrology, Dialysis and Transplantation Children's Mercy, University of Missouri-Kansas City School of Medicine, Kansas City, United States of America.

[3] Division of Pediatrics, Luis Calvo Mackenna Children's Hospital, Faculty of Medicine, University of Chile, Santiago, Chile

[4] Department of Nephrology, Children's Hospital at Westmead, School of Public Health, University of Sydney, Sydney, Australia

[5] Department of Pediatric Nephrology, Emma Children's Hospital AMC, University of Amsterdam, Amsterdam, The Netherlands

[6] Division of Nephrology and Rheumatology, National Center for Child Health and Development, 2-10-1, Okura, Setagaya-ku, Tokyo 157-8535, Japan

[7] Division of Pediatric Nephrology, The Johns Hopkins University School of Medicine, Baltimore, The United States of America.

[8] Pediatric Nephrology Unit, Faculty of Medicine, Cairo University, Cairo, Egypt

[9] Kidney Development & Pediatric Kidney Disease Research Center, Children's Hospital of Fudan University, Shanghai, China

[10] Division of Pediatric Nephrology, Heidelberg University Center for Pediatrics and Adolescent Medicine, Heidelberg, Germany

Keywords: *Registry; Epidemiology; Renal replacement therapy; Dialysis; Kidney transplantation.*

Funded by International Pediatric Nephrology Association

Word Count: 3142 (excluding abstract, references, tables and figures)

Word count abstract: 220

Correspondence to:

Sophie Ploos van Amstel, MD

IPNA Registry, Department of Medical Informatics,

Academic Medical Center, University of Amsterdam

P.O. Box 22700, 1100 DE Amsterdam, The Netherlands

Telephone +31 20 566 5738; E-mail: s.ploosvanamstel@amc.uva.nl

SUPPLEMENTARY MATERIAL:

SEARCH STRATEGY:

Date search: 21st November 2016
Active Filter: Humans

#1 "Search hematuria", 19811
#2 "Search hemoglobinuria",4548
#3 "Search kidney diseases",426164
#4 "Search proteinuria",42016
#5 "Search (renal[Text Word]) OR kidney*[Text Word]",656356
#6 "Search hemodialysis[Text Word]",49783
#7 "Search hemofiltrat*[Text Word]",5371
#8 "Search ((CAPD[Text Word]) OR CCPD[Text Word]) OR APD[Text Word]",7850
#9 "Search dialysis[Text Word]",121956
#10 "Search glomeruloscler*[Text Word]",6942
#11 "Search glomeruloneph*[Text Word]",34639
#12 "Search (((nephritis[Text Word]) OR nephrotic[Text Word]) OR nephrosis[Text Word]) OR nephropath*[Text Word]",81220
#13 "Search proteinuri*[Text Word]",31875
#14 "Search (#1 OR #2 OR #3 OR #4 OR #5 OR #6 OR #7 OR #8 OR #9 OR #10 OR #11 OR #12 OR #13)",769028
#15 "Search (registry[Title]) OR registries[Title]",15517
#16 "Search database[Title]",10821
#17 "Search (#15) OR #16",26172
#18 "Search ((#17) AND #14) NOT editorial[Publication Type]",1264
#19 "Search ((((((Pediatric) OR Pediatric*) OR pediatric) OR pediatric*) OR child*) OR infant) OR adolescent",3435802
#20 "Search (#18) AND #19",380
